# Long-term mucosal injury and repair in a murine model of pelvic radiotherapy

**DOI:** 10.1038/s41598-019-50023-4

**Published:** 2019-09-24

**Authors:** Dilip K. Malipatlolla, Piyush Patel, Fei Sjöberg, Sravani Devarakonda, Marie Kalm, Eva Angenete, Elinor Bexe Lindskog, Rita Grandér, Linda Persson, Andrea Stringer, Ulrica Wilderäng, John Swanpalmer, Hans Georg Kuhn, Gunnar Steineck, Cecilia Bull

**Affiliations:** 1 The Division of Clinical Cancer Epidemiology, Department of Oncology at the Institute of Clinical Sciences, The Sahlgrenska Academy at the University of Gothenburg, Gothenburg, Sweden; 2 Department of Pharmacology at the Institute of Neuroscience and Physiology, The Sahlgrenska Academy at the University of Gothenburg, Gothenburg, Sweden; 3 The Department of Surgery at the Institute of Clinical Sciences, The Sahlgrenska Academy at the University of Gothenburg, Gothenburg, Sweden; 4 School of Pharmacy and Medical Sciences, University of South Australia, Adelaide, Australia; 5 Department of Radiation Physics at the Institute of Clinical Sciences, The Sahlgrenska Academy at the University of Gothenburg, Gothenburg, Sweden; 6 Institute of Neuroscience and Physiology, The Sahlgrenska Academy at the University of Gothenburg, Gothenburg, Sweden; 7 Department of Infectious Diseases at the Institute of Biomedicine, The Sahlgrenska Academy at the University of Gothenburg, Gothenburg, Sweden

**Keywords:** Cell biology, Physiology, Gastrointestinal models

## Abstract

Chronic intestinal injury after pelvic radiotherapy affects countless cancer survivors worldwide. A comprehensive understanding of the long-term injury dynamics is prevented in available animal models. With linear accelerators that are used to treat cancer in patients, we irradiated a small volume encompassing the colorectum in mice with four fractions of 8 Gy per fraction. We then determined the long-term dynamics of mucosal injury, repair, and the duration of inflammation. We show that crypt fission, not cell proliferation, is the main long-term mechanism for rescuing crypt density after irradiation, and provides a potentially wide window for clinical interventions. Persisting macrophage aggregations indicate a chronic mucosal inflammation. A better understanding as to how crypt fission is triggered and why it fails to repair fully the mucosa may help restore bowel health after pelvic radiotherapy. Moreover, anti-inflammatory interventions, even if implemented long after completed radiotherapy, could promote bowel health in pelvic cancer survivors.

## Introduction

Patients who survive cancer through the help of pelvic radiotherapy have a life-long risk of sequelae that result from radiation-induced injury to the intestines. Permanent changes in bowel habits, which can arise months to years, even decades, after completion of the treatment, are estimated to occur in 90% of pelvic cancer survivors^1,2^. In the literature, the symptoms following pelvic radiotherapy are sometimes collectively referred to as radiation-induced survivorship diseases^3^ or pelvic radiation disease^1^. We have previously identified five underlying syndromes (symptom clusters) and labelled them as follows: faecal-leakage syndrome; faecal-urgency syndrome; excessive mucus discharge; excessive gas discharge; and blood discharge^3^. The sensitivity of the gut mucosa to ionising irradiation is linked to the high proliferation rate of stem cells and progenitor cells at the base of the crypts, as these cells expose their DNA to the ionising beams during the S-phase of the cell cycle^4^. In addition, radiation directed towards the intestines is believed to be deleterious to the endothelial cells that supply the mucosa with blood, and the formed small thrombi exacerbate further the vascular injury^5^. Despite reports of successful amelioration of late-occurring symptoms using anti-inflammatory agents, it is commonly believed that the acute inflammation of the mucosa subsides as the tissue moves into a fibrotic stage^6^. However, the long-term trajectory of radiation-induced intestinal injury is difficult to study, since most of the models that simulate clinically relevant doses kill the animal within a week or two^7^. There are models that allow for longer survival times, e.g., those entailing bowel exteriorisation or the shielding of vital organs. Unfortunately, these models either create uncertainty in relation to the delivered dose of radiation, as in the case of shielding, or the procedure itself may interfere with the outcome, as is the case with surgery for bowel exteriorisation. Surgery also prevents repeated administration of radiation doses, e.g., fractionation. By employing linear accelerators that are used in the clinic, accurate photon irradiation of a well-defined target volume can be performed, thereby avoiding off-target effects. The level of uncertainty related to the absorbed dose delivered to the target volume is relatively low. The dose rate is identical to that used for patient treatments, and the radiation can be fractionated^8^. We have recently developed a model of fractionated irradiation of the mouse colorectum using linear accelerators, whereby we deliver clinically relevant doses to the gut mucosa while safeguarding animal survival^9^. The model produces dose- and fraction-dependent effects on the mucosa that are similar to those seen in humans. In the present study, we used the model to determine the dynamics of radiation-induced injury to the colorectal mucosa up to 30 weeks after tissue irradiation, with special emphasis on the repair processes and inflammatory activities. The validity of the murine model was examined in rectal biopsies from patients treated with pelvic radiotherapy.

## Results

### Animal health status

**Figure 1:**
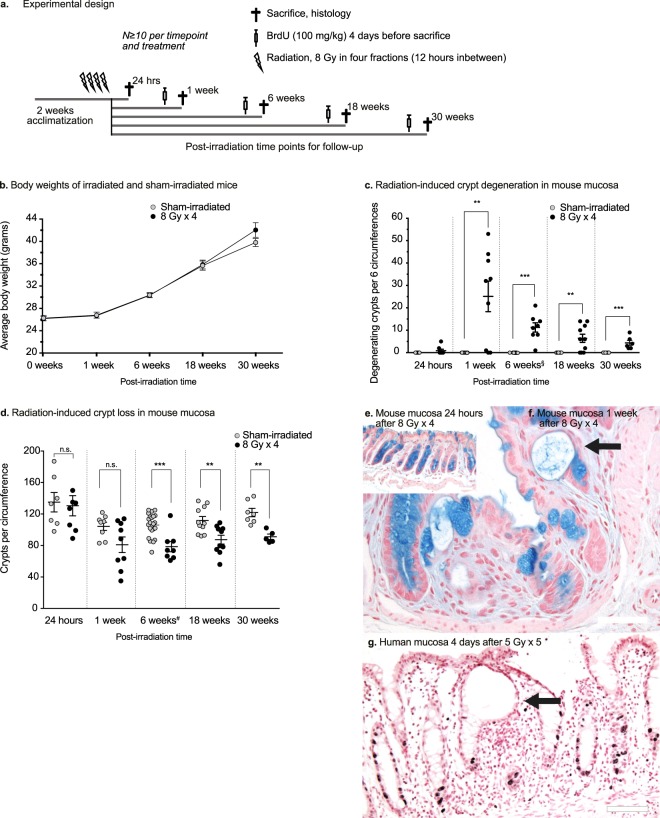
(**a**) Experimental design. Male C57BL/6J mice (N ≧ 10 per treatment and time point) were irradiated with four fractions of 8 Gy (8 Gy × 4) with 12 hours between each delivered fraction. Four days before sacrifice, the mice were injected with BrdU (100 mg/kg), to follow crypt cell survival. Colorectal tissues were harvested at 24 hours, 1 week, 6 weeks, 18 weeks, and 30 weeks post-irradiation. (**b**) Weight curves (grams) for the 30-week group. The mice survived and gained weight in a manner similar to the sham-irradiated controls. At 30 weeks post-irradiation, there is a trend towards increased weight in the irradiated mice. (**c**) Number of degenerating crypts per six circumferences. Radiation-induced crypt degeneration is evident first at 1 week post-irradiation. The number of degenerating crypts diminishes over time. (**d**) Crypt loss over time. Quantification of the surviving crypts per circumference of the colorectum reveals that the crypt loss is permanent. At 24 hours post-irradiation, no crypt loss is detected. One week after irradiation, a few animals have lost more than 50% of their crypts per circumference, as compared to the sham-irradiated controls, while some animals still have the normal number of crypts. At 6 weeks post-irradiation, crypt loss is seen in nearly all the animals in the irradiated group. The irradiated mucosa has approximately 25% fewer crypts than the sham-irradiated mucosa, and this remains unchanged throughout the experiment, despite what appears to be a slight increase in the number of crypts with age in both groups. ^§,#^Data from the 6-week group have been reported previously^26^, and^9^, respectively. (**e**,**f**) Histology of irradiated mucosa samples. There are negligible numbers of degenerating crypts 24 hours after irradiation (**e**). However, at 1 week post-irradiation, several animals have multiple degenerating crypts (**f**, arrow) with few goblet cells (Alcian Blue and Nuclear Fast Red stain). Scale bars, 50 μm. (**g**) Degenerating crypt in the rectal mucosa of a cancer patient 4 days after 5 Gy × 5 (G, arrow). There are multiple dividing cells in nearby crypts, visualised as darkly stained Ki-67^+^ nuclei. Only two Ki-67^+^ cells are visible at the bottom of the degenerating crypt. Scale bar, 100 μm.

All the animals were inspected twice a week and weighed once every week until the end of the study (Fig. 1b). There was no statistically significant difference in body-weight between the irradiated and sham-irradiated mice at any of the time-points. The mice did not exhibit any signs of diarrhoea or constipation, although a few mice exhibited signs of mucus discharge sporadically, with the first observations made at 18 weeks post-irradiation. One mouse died from an unknown cause. Overall, the mice appeared to be healthy throughout the experiment.

### Crypt degeneration

A total of six sections per animal was analysed for degenerating crypts at five different time-points post-irradiation (Fig. 1c). At 24 hours post-irradiation, degenerating crypts were very rarely seen (P = 0.47, as compared to sham-irradiated animals). The highest number of degenerating crypts was found in animals at 1 week post-irradiation (P = 0.004), but the number had declined by more than 50% 5 weeks later (6 weeks; P < 0.001) and by nearly another half at 18 weeks (18 weeks; P = 0.003). Despite the decrease, there remained a considerable number of degenerating crypts at 30 weeks post-irradiation (P < 0.001). Degenerating crypts were not observed at any time-point in any of the sham-irradiated animals.

### Crypt loss

To quantify the radiation-induced loss of crypts over time, the total number of crypts per circumference in two sections per slide was counted in all the groups. At 24 hours (P = 0.81) and 1 week (P = 0.06) post-irradiation, no significant crypt loss was observed, whereas there was statistically significant crypt loss at 6 weeks (P = 0.0002), 18 weeks (P = 0.004), and 30 weeks (P = 0.001) (Fig. 1d–f). Human irradiated rectal mucosa showed similar gross morphological changes with approximately one-third of the biopsies containing enlarged degenerating crypts at 3 to 5 days after completed radiotherapy with 25 Gy in 5 fractions (illustrated in Fig. 1g, data not shown). In the biopsies taken 6 to 11 weeks after 45–50 Gy in 25 fractions, only one biopsy showed crypt degeneration. Crypt degeneration was not observed in the any of the non-irradiated biopsies, consistent with our findings in mice where we have never observed crypt degeneration in sham-irradiated mucosa.

### Inflammatory activity

**Figure 2:**
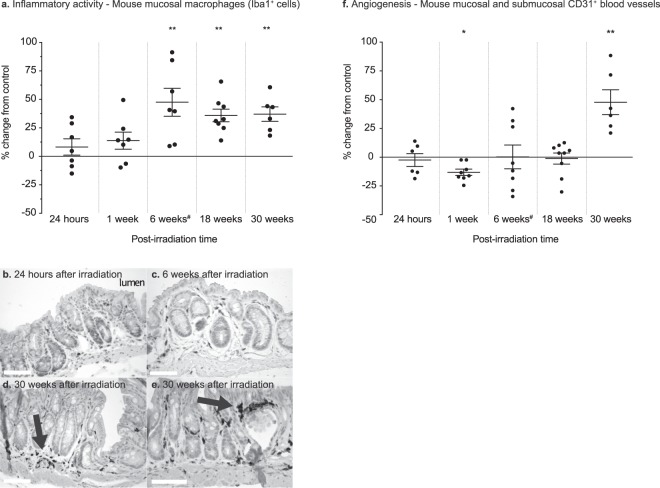
(**a**) Percentage change (relative to sham-irradiated animals) in the number of Iba1^+^ mucosal macrophages at 24 hours, 1 week, 6 weeks, 18 weeks, and 30 weeks post-irradiation. An increase in infiltrating macrophages is not seen until 6 weeks after irradiation, with most of the animals exhibiting an increase in the number of mucosal macrophages. This remains unchanged over time, indicating a state of long-lasting, possibly chronic mucosal inflammation. (**b**–**e**) Iba1^+^-expressing macrophages visualised with DAB immunohistochemistry in the irradiated colorectal mucosa at various time-points after irradiation. Macrophages are still abundant in the mucosa 30 weeks after irradiation and tend to aggregate around the blood vessels, close to the degenerating crypts, and under the epithelial surface (arrows). Scale bars, 50 μm. (**f**) CD31^+^ blood vessels in irradiated animals, shown in terms of the percentage change relative to the sham-irradiated animals. The increase in blood vessels at 30 weeks after irradiation suggests late, compensatory angiogenesis. ^#^The data for the 6-week group have been published previously^9^.

To determine the trajectory of inflammatory activity, we quantified the numbers of macrophages at all five time-points (Fig. 2a). At 24 hours post-irradiation, the average number of macrophages overall did not differ significantly between the irradiated and sham-irradiated animals (P = 0.63). At one week post-irradiation the average abundance of macrophages had increased slightly in the irradiated animals (P = 0.13), while at six weeks post–irradiation the difference between sham-irradiated and irradiated was pronounced (P = 0.003). At 18 weeks (P = 0.003) the number of macrophages stabilised and the high abundance of macrophages persisted in the 30-week cohort (P = 0.003) (Fig. 2a–e).

### Angiogenesis

There were no significant differences in the number of blood vessels compared with the sham-irradiated animals at 24 hours (P = 0.80), 6 weeks (P = 0.98), and 18 weeks (P = 0.48) post-irradiation. Significant loss of blood vessels was observed at 1 week (P = 0.04) post-irradiation, as compared to the controls, and at 30 weeks post-irradiation there was a significant increase in the number of blood vessels (P = 0.001) (Fig. 2f).

### Crypt fission

**Figure 3:**
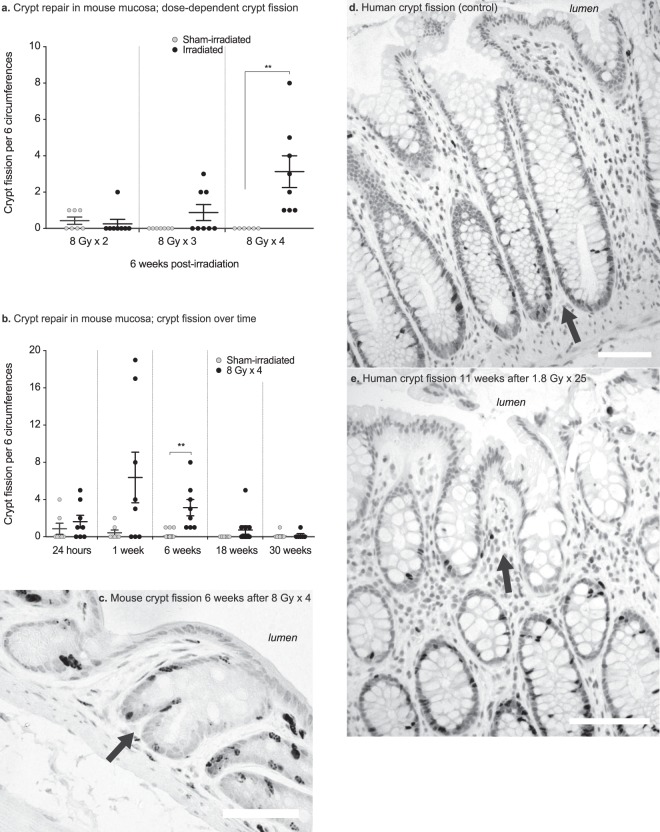
(**a**) Numbers of crypt fission events per 6 circumferences after different fractionation schedules. The occurrence of crypt fission is dose-dependent, as seen 6 weeks after irradiation. (**b**) Number of crypt fission events per six circumferences at 24 hours, and at 1 week, 6 weeks, 18 weeks, and 30 weeks after irradiation or sham-irradiation. A few of the irradiated animals show many crypt fission events 1 week after irradiation, and at 6 weeks post-irradiation all the animals exhibit crypt fission; thereafter the number of fission events declines. At 30 weeks post-irradiation, there is no difference in the number of crypt fissions between the irradiated and sham-irradiated animals. (**c**) Irradiated mouse colorectal mucosa 6 weeks after 8 Gy × 4 of irradiation. The dark nuclei are cells that are labelled with BrdU, showing newly born cells migrating to form two new crypt walls at the crypt mid-line (arrow). (**d**) Crypt fission in a non-irradiated human rectal biopsy (arrow), showing multiple darkly stained Ki-67^+^ proliferating cells at the bottom of the dividing crypt. (**e**) Crypt fission in a human rectal biopsy (arrow) harvested 11 weeks after 1.8 Gy × 25. A few Ki-67^+^ proliferating cells are seen at the bottom of one of the two resulting crypts.

We quantified crypt fission 6 weeks after the application of 2, 3 or 4 fractions of 8 Gy each. Crypt fission increased in dose- and fraction-dependent manners and was significantly increased in the animals that received 4 fractions of irradiation (Fig. 3a; 8 Gy × 2, P = 0.6; 8 Gy × 3, P = 0.09; and 8 Gy × 4, P = 0.01, as compared to sham-irradiated animals). To monitor the regenerative response, we also quantified crypt fission at all other time-points (Fig. 3b). At 24 hours post-irradiation, no regenerative response in terms of crypt fission could be observed (P = 0.42, as compared to the sham-irradiated animals). At 1 week post-irradiation, very high levels of crypt fission were found in a few animals, while some animals did not display any crypt fission yet (P = 0.07). At 6 weeks post-irradiation, all the irradiated animals displayed crypt fission (P = 0.01). At 18 and 30 weeks post-irradiation, the regenerative response had subsided and crypt fission was back to the control levels (P = 0.14 and P = 0.84, respectively). Crypt fission events were also observed in the human rectal biopsies from both treatment groups and the non-irradiated subjects (Fig. 3d,e).

### Rates of cell proliferation and cell survival in individual crypts after irradiation

**Figure 4:**
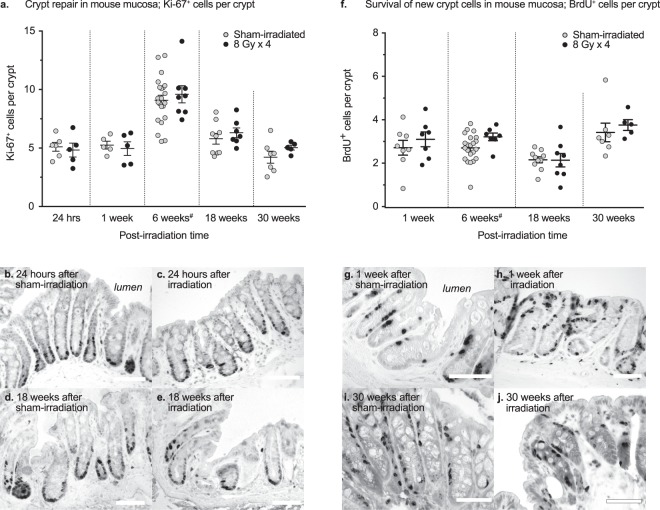
(**a**) No statistically significant change in the numbers of Ki-67-labelled cells is observed after irradiation at any of the time-points chosen. (**b**–**e**) Numbers of Ki-67^+^ cells at 24 hours (**b**,**c**) and 18 weeks (**d**,**e**) post-irradiation, showing similar numbers of proliferating cells in the surviving crypts of irradiated animals, as compared to sham-irradiated animals. (**f**) Percentage change (relative to sham-irradiated animals) in the number of surviving cells per crypt, as evidenced by BrdU-labelling. The rate of cell survival remains unchanged at 24 hours after irradiation and thereafter. (**g**–**j**) BrdU^+^ crypt cells in irradiated and sham-irradiated mucosal samples after 1 week and 30 weeks. Scale bars, 50 μm. ^#^Data for the 6-week group have been previously published^9^.

To determine the effect of irradiation on crypt cell proliferation over time, we quantified the number of newly produced crypt cells using the proliferation marker Ki-67 at the various time-points (Fig. 4a–e). No statistically significant differences in cell proliferation were observed between the sham-irradiated and irradiated mice at 24 hours (P = 0.69), 1 week (P = 0.67), 6 weeks (P = 0.54), 18 weeks (P = 0.43) and 30 weeks (P = 0.23) post-irradiation.

To determine the survival of newly created crypt cells in response to radiation injury, mice were injected with BrdU 4 days before sacrifice, except for the 24-hour animals. The survival rates of the newly born crypt cells were unaffected at all the examined time-points of 1 week (P = 0.44), 6 weeks (P = 0.07), 18 weeks (P = 0.95) and 30 weeks (P = 0.55) post-irradiation (Fig. 4f–j).

## Discussion

In commonly used models of radiation-induced injury to the gut mucosa, off-target effects from large radiation fields and the toxicity of higher doses of radiation for the intestines hinder long-term studies^7^. Our model shows that infiltrating macrophages do not appear in the mucosal tissue until several weeks after the initial injury. However, once they have invaded the tissue, the macrophages persist for a long time, possibly rendering the condition chronic. Contrary to expectations and despite widespread crypt loss, we did not observe any changes in the production or survival of newly produced crypt cells, which might signal a late repair response. Instead, the regenerative activities of the mucosa were reflected in increased rates of crypt fission, a phenomenon that was both time- and fraction-dependent and lasted for many weeks after the initial insult. The occurrence of crypt degeneration and crypt fission in irradiated human rectal tissues was confirmed.

Following irradiation of the colon, an acute inflammatory response is triggered in the mucosa, involving the production of reactive oxygen species and the release of cytokine and chemokine signals from damaged cells in the epithelium, endothelium, and directly from the extracellular matrix. Mucosal infiltration of immunomodulating cells after irradiation of the colon is also considered to be an early response^10^. We used the pan-macrophage marker Iba1 to identify macrophages in the mucosa. While this marker may also be expressed in a small subset of dendritic cells, it is widely expressed in colonic macrophages, which means that the majority of our Iba1^+^ cells are expected to be macrophages^11,12^. We found an increase in the number of macrophages in the mucosa first at 6 weeks post-irradiation, which was delayed relative to the peak of crypt loss at 1 week post-irradiation. This suggests that the macrophages are involved in regenerative processes, fibrotic mechanisms or other damage control processes, e.g., the phagocytosis of antigens, rather than the acute inflammatory response. At the two latest time-points studied, all the irradiated animals had increased macrophage numbers in their mucosal tissues compared to the controls. We found aggregations of macrophages in areas where “gut leakiness” might be suspected, such as right under the epithelial border where the crypts had been lost, near degenerating crypts, and around blood vessels. Therefore, we propose that the irradiated mucosa is permeable to pathogens long after the irradiation has ceased, and that the leakiness of the barrier attracts macrophages^13^. Although macrophages can promote intestinal barrier function and homeostasis, the presence of macrophages underneath the epithelial layer can also increase the extent of leakiness^14^. Furthermore, macrophages internalise extracellular matrix components at a high rate, thereby contributing to tissue degradation and remodelling, and even tumour invasion^15^. The reasons for the long-lasting persistence of the macrophages in the irradiated mucosa, and the effects thereof, remain to be defined. Importantly, the demonstration of similar long-term persistence of macrophages in human irradiated mucosa would lend support to the notion that pelvic radiotherapy, after the acute phase, induces a mild but chronic inflammatory activity in the irradiated volumes of the gut mucosa. While this type of sub-clinical inflammatory activity might go unnoticed in the clinical setting, it could nevertheless promote and maintain intestinal disease in pelvic cancer survivors.

Potten and co-workers measured crypt cell apoptosis and found it that it peaked at around 4 hours post-irradiation^4^. In contrast, crypt degeneration appears to be a much slower process. We observed few degenerating crypts 24 hours after the last fraction of irradiation, which corresponded to 60 hours after delivery of the first fraction. One week after the last fraction was delivered, there was a sharp increase in the number of degenerating crypts. The loss of crypts appeared to be permanent, since the crypt density in the irradiated mucosa never recovered.

Regeneration of the mucosa can progress through two linked albeit separate mechanisms: crypt cell proliferation and crypt fission. Crypt fission proceeds through the division of a crypt along its vertical axis, beginning at the base of the crypt, and results in two daughter crypts^16^. Besides being a key player in natural growth, crypt fission is believed to be a response to crypt loss^17^. We observed, as a profound response to irradiation, a several-fold increase in the rate of crypt fission. Events of crypt fission were also seen in the colorectal biopsy from a patient eleven weeks after completed radiotherapy. Although natural crypt fission was noted in a few of our younger sham-irradiated control animals, the phenomenon was rarely seen in the 7- and 10-months-old animals (18 and 30 weeks after sham-irradiation). In contrast, irradiation triggered crypt fission in dose- and fraction-dependent manners. Park and colleagues showed that crypt fission in the colon can occur without a significant increase in cell proliferation^18^, and they suggested that crypt fission in the colon can be prompted by other factor(s). It is possible that the process of degeneration signals nearby crypts to undergo fission. However, we were unable to detect a direct correlation between the levels of crypt degeneration and crypt fission (data not shown).

Historically, studies of radiation-induced injury of the gut have focussed on crypt cell proliferation, rather than crypt fission. There are reports of a lasting loss of crypt cell proliferation, as well as compensatory increases in cell proliferation rates after irradiation^19,20^. Many agents have been reported that either stimulate or inhibit the regenerative response after irradiation via alterations of cell proliferation in the crypt stem cell niche^21–23^. We found no significant differences in cell proliferation or cell survival between the irradiated animals and sham-irradiated controls at any of the chosen time-points, apart from the acute cell death at 4.5 hours post-irradiation that we previously reported^9^. The discrepancies between our findings and those of other studies concerning cell proliferation may be due to the use of different exclusion or inclusion criteria when quantifying the parameter. We employed strict inclusion criteria and only counted those crypts that were open towards the lumen and cut along their perpendicular axis, and we did not include crypts that were undergoing degeneration. We believe that Ki-67^+^ cells are lost within hours after irradiation, however if the crypt is not entirely sterilised in the subsequent fractions surviving crypts “mask” the injury to the stem cell niche by maintaining normal proliferation rates until the entire stem cell pool is depleted. When that happens, the crypt degenerates. Another possible explanation for the discrepancies between our findings and those of others concerning cell proliferation rates is that the small radiation field produced by the linear accelerators minimises the risk of off-target effects, which could indirectly influence cell proliferation. Lastly, there are differences in growth mechanisms between the colon and the small intestine, such that in a healthy state the colon depends mainly on crypt fission for growth, whereas the small intestine depends primarily on crypt cell proliferation^24^. Thus, it is possible that the regenerative response in the far less studied colon differs from the regenerative response in the small intestine.

There are diverging opinions as to whether the acute, primary lesion caused by radiation to the mucosa involves injury to the highly proliferative crypt stem cells or injury to the endothelium^7,17^. Similar to the trajectory of crypt degeneration, the numbers of CD31^+^ vessels in the mucosa and submucosa were unchanged at 24 hours post-irradiation, whereas there was a moderate reduction in the numbers of CD31^+^ blood vessels at 1 week post-irradiation. The loss of CD31^+^ blood vessels was transient, with the numbers increasing by nearly 50%, as compared to the sham-irradiated controls, at 30 weeks post-irradiation. The larger arterioles that we quantified supply the crypts with blood via the peri-cryptal microvasculature^25^. If the peri-cryptal microvasculature is damaged beyond repair, there may be an underlying chronic hypoxia, which could accelerate stem cell depletion and crypt loss and trigger compensatory angiogenesis.

Based on the findings presented here, we suggest that irradiation of the colorectum causes an inflammation that is not resolved by the time that the lost crypts have been replaced by fibrotic tissue. If this can be confirmed in pelvic cancer survivors who have persistent bowel dysfunction, a successful approach to restoring bowel health in these patients could take into account the ongoing intestinal inflammation. Furthermore, we suggest that long-term repair processes in the colorectum after irradiation are manifested through the largely overlooked phenomenon of crypt fission, rather than stem cell or progenitor cell proliferation. Since crypt fission events were found at all of the time-points studied after the initial insult, we believe that the window of opportunity for mucosal healing after radiotherapy is much wider than is commonly assumed. Successful repair via crypt fission may predict both the long-term prognosis of bowel health after pelvic radiotherapy and the outcomes of protective interventions. Thus, studies that give us a deeper understanding of how crypt fission is regulated and why it fails to restore completely the damaged mucosa are warranted.

## Methods

All methods were carried out in accordance with relevant guidelines and regulations.

### Human biopsies

Biopsies from a total of 32 patients who had received either 25 Gy in 5 fractions (5 Gy × 5, N = 19) as preoperative irradiation for rectal cancer followed by surgery 3 to 5 days after completion of the radiotherapy, or 45–50 Gy in 25 fractions (1.8–2 Gy × 25, N = 13) with concomitant capecitabine followed by surgery 6 to 11 weeks after completion of radiotherapy, were inspected for the occurrence of crypt degeneration and/or crypt fission. The biopsies were collected at time of surgery from macroscopically normal-appearing mucosa located approximately 10 cm from the tumour. Biopsies from 13 rectal cancer patients who had not received irradiation were also inspected. The biopsies were fixed in paraformaldehyde and embedded in paraffin prior to sectioning. The local Ethics Committee of the University of Gothenburg approved the study (EPN 118–15) and informed consent was obtained from the patients.

### Animals

The 7–8-week-old male C57BL/6J mice were purchased from Charles River Laboratories GmbH (Sulzfeld, Germany) and maintained at a constant temperature (20 °C) with 42% relative humidity and a 12-hour day/light cycle. The mice had free access to food and water. All the experimental animal procedures were approved by the Gothenburg Committee of the Swedish Animal Welfare Agency (Application number 22–2015).

### Irradiation procedure

The method has been described in detail elsewhere^9^. In brief, at 9 to 10 weeks of age, the mice were anaesthetised and irradiated using a linear accelerator (Varian TrueBeam, Varian Medical Systems Inc., Charlottesville, VA, USA) with 6 MV nominal photon energy. The irradiation itself is pain-free but anaesthesia was used to immobilize the mice during the procedure. Mice were anaesthetised under the linear accelerator using a portable anaesthesia unit (Univentor 410 Anaesthesia Unit, Univentor Limited, Malta) with an air pump and a nose mask delivering a continuous air flow (300 mL/min) of 2.5–3% isoflurane. A silicone mold ensured identical positioning of the mice and the radiation field was oriented so that approximately 1.5 cm of the distal bowel was irradiated. Care was taken to exclude the spinal cord and testicles from the radiation field. Four fractions of 8 Gy per fraction (8 Gy × 4) were delivered at a dose-rate of 5.9 Gy/min. The fractions were spaced at 12 hours. The dose variation within the target volume was estimated to be ± 5%. Sham-irradiated mice were placed under the linear accelerator but received only anaesthesia.

### Experimental design

An overview of the experimental design is shown in Fig. 1a. For each time point, four cages with five mice in each were randomly allocated to either irradiation or sham-irradiation (N = 10 per treatment, except for the 6-week time point that had a larger control group). Sacrifice after the last fraction occurred at 24 hours (acute), 1 week (early), 6 weeks (early intermediate), 18 weeks (late intermediate) or 30 weeks (late). Four days before sacrifice, all the mice, except those belonging to the acute (24-hour) group, were given a single injection of BrdU (100 mg/kg; Sigma-Aldrich), to determine the rate of survival of proliferating cells. Tissue that was broken or fragmented during harvesting and tissue processing or poorly stained during histochemistry were excluded, and the final number of animals analysed after tissue processing are presented in Table 1 (under column “N”).

**Table 1:** Descriptive statistics.

	Fig	24 hours						1 week					
		Sham-irradiation			8 Gy × 4			Sham-irradiation			8 Gy × 4		
		Mean	+SEM	N	Mean	±SEM	N	Mean	± SEM	N	Mean	±SEM	N
	Fig	6 weeks						18 weeks					
		Sham-irradiation			8 Gy × 4			Sham-irradiation			8 Gy × 4		
		Mean	±SEM	N	Mean	±SEM	N	Mean	±SEM	N	Mean	±SEM	N
	Fig	30 weeks											
		Sham-irradiation			8 Gy × 4								
		Mean	±SEM	N	Mean	±SEM	N						
Crypts/circumference	1d	135,0	12,3	7	131,0	12,8	8	104,4	5,2	8	80,9	9,9	9
Degenerating crypts	1c	0,0	0,0	7	0,9	0,6	8	0,0	0,0	8	25,1	6,8	9
Crypt fisson	3b	0,9	0,6	7	1,6	0,7	8	0,4	0,3	7	6,4	2,7	8
Ki-67^+^ cells per crypt	4a	5,1	0,4	6	4,8	0,6	5	5,3	0,3	5	5,0	0,6	5
BrdU^+^ cells per crypt	4f	N/A	N/A	N/A	N/A	N/A	N/A	2,7	0,3	8	3,1	0,3	7
CD31^+^blood vessels/circumference	2f	44,9	2,6	6	46,1	3,4	8	54,3	2,8	7	47,2	1,5	8
Iba1^+^ cells/mm^2^	2a	1587,0	46,4	6	1717,0	114,0	7	1255,0	49,0	7	1428,0	94,1	7
Crypts/circumference	1d	105,9	3,2	22	78.5	6,5	8	111,8	5,0	11	87,4	5,6	10
Degenerating crypts	1c	0,0	0,0	22	11,3	2,1	8	0,0	0,0	10	6,4	1,8	10
Crypt fisson	3b	0,0	0,0	6	3,1	0,9	8	0,0	0,0	10	0,7	0,4	11
Ki-67^+^ cells per crypt	4a	9,1	0,4	22	9,6	0,7	8	5,8	0,5	9	6,3	0,4	7
BrdU^+^ cells per crypt	4 f	2,7	0,1	22	3,2	0,2	7	2,2	0,1	10	2,1	0,3	8
CD31^+^blood vessels/circumference	2 f	38,0	2,7	19	38,1	3,9	8	22,2	1,1	10	20,9	1,4	10
Iba1^+^ cells/mm^2^	2a	1124,9	77,3	24	1659,3	138,3	7	1367,0	108,5	10	1858,0	76,4	8
Crypts/circumference	1d	121,8	5,3	7	91,0	3,4	5						
Degenerating crypts	1c	0,0	0,0	8	4,3	1,1	6						
Crypt fisson	3b	0,1	0,1	8	0,2	0,2	6						
Ki-67^+^ cells per crypt	4a	4,2	0,5	7	5,0	0,2	5						
BrdU^+^ cells per crypt	4f	3,4	0,4	7	3,8	0,2	5						
CD31^+^blood vessels/circumference	2f	32,6	1,6	7	48,3	3,5	6						
Iba1^+^ cells/mm^2^	2a	1255,0	91,9	8	1720,0	79,6	6						

### Tissue collection

Each mouse was deeply anaesthetised with isoflurane (Isoba® vet; Schering-Plough Animal Health, Harefield, Uxbridge, Middlesex, England), the abdomen was opened, and the colon was flushed with paraformaldehyde (Histofix; Histolab Products AB, Askim, Sweden). Thin, soft silicone tubing (OD, 1.19 mm and ID, 0.64 mm; AgnThos AB, Lidingo, Sweden) was carefully inserted rectally to preserve the tissue shape during fixation. Then, an approximately 1 cm piece of colorectal tissue (from the anus and proximally) with silicone tubing was excised and fixed in Histofix for 24 hours at +4 °C. The samples were transferred to 70% ethanol overnight and the silicone tubing was carefully removed before dehydration and paraffin embedding.

### Serial sectioning

Sections (4-μm-thin) were cut on a microtome (Leica RM2235; Leica Biosystems) and mounted on slides in a 1:6 series. Thus, each section on a slide was separated from the previous section by at least 20 μm, preventing analysis of the same crypt twice in adjacent sections. The human biopsies were cut on a micotome in 6-μm-thin sections.

### Immunohistochemistry

After dewaxing in xylene and rehydration in an alcohol series, the sections were boiled in citrate buffer (pH 6) in a pressure cooker for 3 minutes for antigen retrieval, followed by incubation in 0.6% H_2_O_2_ to inactivate endogenous peroxidase activity. For BrdU detection, the tissue was additionally pre-treated with 2 N HCl followed by borate buffer (pH 8.5). Sections were then incubated with primary antibodies in blocking buffer (TBS containing 0.1% Triton X-100 and 3% donkey serum) either overnight at +4 °C or for 1 hour at room temperature. The antibodies used were as follows: anti-mouse BrdU (1:500 dilution; DAKO); anti-rabbit Ki-67 (1:150; Merck Millipore); goat anti-CD31 (1:150; R&D Systems, Minneapolis, MN, USA), and anti-rabbit Iba1 (1:2000; Wako Industries). This was followed by the addition of the appropriate biotinylated secondary antibody (1:250; Vector Laboratories, Burlingame, CA, USA). After incubation with avidin-biotin solution (Vectastain ABC Elite kit; Vector Laboratories), the antigen was visualised by developing in DAB (Saveen Werner AB, Malmö, Sweden). The sections were then dehydrated in an alcohol series, cleared in xylene, and cover-slipped in Xtra-kitt mounting medium (Medite GmbH, Burgdorf, Germany). The human biopsy samples were treated in a similar way.

### Histochemistry

For visualising the mucosal crypts, either Alcian Blue combined with Nuclear Fast Red or Verhoeff’s Elastic stain was used. For Alcian blue staining, the sections were dewaxed, rehydrated, treated with acetic acid for 3 minutes, and then stained in 1% Alcian Blue 8GX with 3% acetic acid (Histolab AB) for approximately 30 seconds, and rinsed in tap-water. The sections were then stained with Nuclear Fast Red (Histolab AB) for 5 minutes, rinsed once more in tap-water, dehydrated through an alcohol series, and cover-slipped in Xtra-kitt mounting medium. Staining with Verhoeff’s Elastic stain was performed by immersing the sections in Verhoeff’s solution, rinsing in tap-water and thereafter in distilled water, and differentiating in 2% ferric chloride solution. This was followed by the addition of a 5% sodium thiosulfate solution, further rinses in tap-water and distilled water, followed by counterstaining with Van Gieson’s stain, dehydration in an alcohol series, and clearing in xylene before mounting and cover-slipping with Pertex (Histolab AB).

### Crypt numbers

The number of crypts was quantified using a Leica DM6000B microscope (Leica Microsystems AB, Wetzlar, Germany) equipped with a semi-automated stereology system (Stereo Investigator 6; MBF Bioscience Inc., Williston, VT, USA). All the crypts in three whole colorectal circumferences located 48 μm apart were counted per subject, and the average number of crypts per subject was calculated.

### Assessment of crypt fission

Sections that were stained with Alcian Blue/Neutral Fast Red or Verhoeff’s Elastic stain were used for the assessment of crypt fission. For each animal, a total of six sections spaced 24 μm apart was analysed at 40 × magnification. Crypt fission was defined as a crypt with a single opening to the lumen, dividing from the crypt bottom and up along its vertical axis to form two crypts.

### Quantification of proliferating cells

To quantify the number of proliferating cells in the crypts, we only included those crypts that were cut at a perpendicular angle, such that the maximum length of the crypt axis becomes visible with a clear opening towards the lumen. The average number of darkly stained Ki-67-positive cells per crypt was quantified from a total of 24 crypts from two sections located 72 µm apart.

### Quantification of crypt cell survival

To determine the effect of irradiation on the survival of newly produced crypt cells, mice were given a single intra-peritoneal injection of BrdU (100 mg/kg; Sigma-Aldrich, St. Louis, MO, USA) 4 days before sacrifice. BrdU is incorporated into the DNA strands of cells that are dividing at the time of injection, such that the progeny can be tracked over time. For quantifying BrdU-positive cells, the strategy described above for analysing Ki-67-positive cells was used. The average number of BrdU-positive cells per crypt, derived from a total of 24 crypts from two sections spaced 72 μm apart, was calculated.

### Assessment of CD31^+^ blood vessels

An antibody directed against the CD31 antigen was used to label the blood vessels in the colonic mucosa and the submucosal layer. All the vessels in the mucosa and submucosal layers of the colon from two sections located approximately 72 μm apart were counted.

### Quantification of macrophages

The mucosa was analysed for the presence of the pan-macrophage marker ionised calcium-binding adapter molecule 1 (Iba1) in two colorectal sections located 72 μm apart. Using a Leica DM6000B microscope equipped with a semi-automated stereology system, a virtual frame was placed at 0°, 90°, 180° and 270° in relation to the colorectal circumference. The contour of the mucosa between the crypts was traced within each frame, providing a measure of the mucosal surface area. All the Iba1^+^ cells within each traced area (a total of eight areas per animal) were then counted. The number of macrophages per mm^2^ was then calculated by dividing the total number of macrophages counted in both sections by the total area analysed.

### Statistical analysis

For comparing two groups, we used a two-tailed unpaired Student’s *t*-test. All the results are presented as means (±S.E.M). The results were considered to be statistically significant at a P-value ≤ 0.05. For the Ki-67 and BrdU experiments, differences in baseline values required normalisation of some groups for visualisation purposes. The data that were normalised towards the control baseline value were the BrdU data for the 6-week group and the Ki-67 data for the 18-week group. Descriptive statistics are presented in Table 1.

## Data Availability

The datasets generated during and/or analysed during the current study are available from the corresponding author on reasonable request.
